# Body Surface Area-Weighted Left Ventricular Ejection Fraction Enhances Prediction Accuracy of OPCABG Outcomes: A Large Multi-Center Cohort Study

**DOI:** 10.31083/RCM26681

**Published:** 2025-11-26

**Authors:** Zhipeng Wei, Zhihui Zhu, Yuehuan Li, Chenyu Li, Nan Liu, Jiakai Lu, Mingying Wu, Huaibin Wang, Dong Xu, Yu Chen, Yongqiang Lai, Haibo Zhang

**Affiliations:** ^1^Department of Cardiovascular Surgery, Beijing Anzhen Hospital, Capital Medical University, 100029 Beijing, China; ^2^Department of Medicine, Renal Electrolyte and Hypertension Division, Perelman School of Medicine, University of Pennsylvania, Philadelphia, PA 19104, USA; ^3^Center for Cardiac Intensive Care, Beijing Anzhen Hospital, Capital Medical University, 100029 Beijing, China; ^4^Department of Anesthesiology, Beijing Anzhen Hospital, Capital Medical University, 100029 Beijing, China; ^5^Department of Cardiovascular Surgery, Beijing Tongren Hospital, Capital Medical University, 100176 Beijing, China; ^6^Department of Cardiovascular Surgery, Beijing Hospital, 100005 Beijing, China; ^7^Department of Cardiovascular Surgery, Xuanwu Hospital Capital Medical University, 100053 Beijing, China; ^8^Department of Cardiovascular Surgery, Peking University People's Hospital, 100044 Beijing, China

**Keywords:** BSA, LVEF, OPCABG, outcomes

## Abstract

**Background:**

We hypothesized that body surface area (BSA)-weighted left ventricular ejection fraction (LVEF) (bLVEF) would represent a superior predictor of mortality in off-pump coronary artery bypass grafting (OPCABG) patients than standard predictors. LVEF is associated with worse outcomes upon OPCABG, while referring left ventricular measurements to BSA should improve predictability.

**Methods:**

The bLVEF was calculated by multiplying the LVEF by the BSA. The primary endpoint was all-cause mortality within 30 days of hospitalization, while secondary endpoints included major postoperative complications.

**Results:**

A total of 7927 patients from five leading cardiac centers participating in the Chinese Cardiac Surgery Registry were included in the final analysis, of which 7093 (89.48%) had normal LVEF, 639 (8.06%) presented heart failure with mid-range ejection fraction (HFmrEF), and 195 (2.46%) exhibited heart failure with reduced ejection fraction (HFrEF). The average bLVEF in the cohort was 109.63 ± 18.16. Both the mortality (odds ratio (OR) 0.97) and secondary endpoints (OR 0.97) followed a similar trend with increasing bLVEF, indicating that bLVEF is a more reliable predictor of adverse outcomes. The individual components of bLVEF, including BSA (area under the curve (AUC) 0.63) and LVEF (AUC 0.64), made minor contributions to mortality risk with relatively low AUC values. However, these components were less impactful than bLVEF (AUC 0.70). Notably, patients with a bLVEF less than 85 had an increased mortality risk relative to those whose bLVEF was 85 or higher (adjusted OR 4.65 (95% confidence interval (CI): 3.81–5.83; *p* < 0.01)).

**Conclusion:**

The bLVEF serves as a key predictor of mortality in OPCABG patients, effectively eliminating BSA-related bias and demonstrating a strong capacity to predict mortality.

**Clinical Trial Registration:**

NCT02400125, https://www.clinicaltrials.gov/study/NCT02400125.

## 1. Introduction

In the 1990s, interest in performing coronary artery bypass grafting (CABG) on a beating heart, avoiding cardiopulmonary bypass, experienced a resurgence, leading to renewed focus on off-pump surgery [1, 2]. Off-pump coronary artery bypass grafting (OPCABG) has been widely utilized in patients with heart failure (HF) due to its association with reduced perioperative complications and improved long-term outcomes compared to on-pump procedures [3, 4]. The left ventricular ejection fraction (LVEF) is an indicator to evaluate HF, which associates with the outcomes in cardiac surgery [5]. Patients with low LVEF face a high risk of perioperative complications, adversely affecting both immediate and long-term survival. This risk is particularly pronounced in those with ventricular contractility impairment following cardiopulmonary bypass and cardioplegia, regardless of other comorbidities [6, 7]. In OPCABG, reduced LVEF, often associated with ischemic cardiomyopathy, is linked to an increased risk of long-term complications [8, 9, 10]. Further investigation is needed to elucidate the relationship between LVEF and perioperative outcomes in OPCABG, offering greater insight into its prognostic significance.

LVEF refers to the percentage of blood volume in the left ventricle at the end of diastole that is pumped out with each contraction, which can be expressed mathematically as $LVEF\left(\%\right)=\frac{\text{ Stroke volume }}{\text{ End-diastolic volume }}$. End-diastolic volume is influenced by several factors, including a patient’s weight, body mass index (BMI), and body surface area (BSA), the latter being a parameter calculated based on height and weight [11]. BSA is positively correlated with blood pressure and serves as a fairly accurate indicator of total body water. It is commonly used to normalize cardiac output to cardiac index and to estimate the glomerular filtration rate (GFR) [12, 13, 14, 15]. In this context, we propose normalizing the end-diastolic volume by BSA to reduce its bias on LVEF. The resulting BSA-adjusted LVEF (bLVEF) is calculated as the stroke volume divided by the normalized end-diastolic volume ($bLVEF\left(\%\right)=\frac{\text{ Stroke volume }}{\text{ End-diastolic volume }\,\left(\frac{1}{\mathrm{BSA}}\right)}$). This study aims to investigate the roles of LVEF, BSA, and bLVEF in a special clinical setting and to evaluate whether their interactions following OPCABG can improve patient-centered care.

Consequently, we aimed to explore the distinct effects of LVEF, BSA, and bLVEF on early clinical outcomes for OPCABG patients using data from five leading Chinese cardiovascular centers. The objectives were to investigate (1) the impact of LVEF on perioperative outcomes, (2) the correlation between BSA and LVEF, and (3) the association of bLVEF with perioperative complications and mortality rates among.

## 2. Materials and Methods

### 2.1 Study Setting and Population

We analyzed data from 7927 patients across five prominent Chinese cardiovascular centers: Beijing Anzhen Hospital, Beijing Tongren Hospital, Beijing Hospital, Peking University People’s Hospital, and Beijing Xuanwu Hospital. The data, sourced from the Chinese Cardiac Surgery Registry database, cover admissions that occurred between December 2016 and January 2021 (refer to **Supplementary Fig. 1**). Clinical data were collected in accordance with the Society of Thoracic Surgeons National Adult Cardiac Database (http://www.sts.org). Data reliability and comprehensiveness were ensured through established procedures, as detailed in prior publications [16]. The study protocol was approved by the Ethics Committee of Fuwai Hospital (Approval No: 2017-943) and is registered at http://www.clinicaltrials.gov (NCT02400125). Patient confidentiality was maintained by pseudo-anonymizing all data, substituting patient names with identification codes and removing private information before analysis. A data committee from the Peking University Clinical Research Institute was responsible for assessing data quality and overseeing data quality and collection. All participants received standard care, with no additional interventions, as previously described [16]. Heart failure was classified according to the European Society of Cardiology, including heart failure with reduced ejection fraction (HFrEF, LVEF <40%) and heart failure with mid-range ejection fraction (HFmrEF, LVEF 40–49%).

### 2.2 Predictor and Outcomes

Patient demographics and clinical features were collected and assessed, including medical histories of peripheral vascular disease, cerebrovascular events, prior myocardial infarctions, previous percutaneous coronary interventions, and New York Heart Association (NYHA) classification. Peoperative test results for serum creatinine, total cholesterol, low-density lipoprotein, blood glucose levels, and estimated glomerular filtration rate (eGFR) were also recorded. Intraoperative echocardiogram data were examined for left ventricular end-diastolic volume (LVED), left ventricular end-diastolic diameter (LVEDd), and left atrial dimension (LAD). Data on concomitant cardiac medications—including nitrate lipid drugs, catecholamines, β-blockers, angiotensin-converting enzyme inhibitors (ACEI), angiotensin receptor blockers (ARB), statins, aspirin, clopidogrel, and ticagrelor—were meticulously documented. The primary outcome was in-hospital all-cause mortality within 30 days, while secondary outcomes included severe postoperative complications such as the postoperative use of extracorporeal membrane oxygenation (ECMO), multiorgan failure, intra-aortic balloon pump (IABP) postoperative usage, postoperative strokes, and myocardial infarctions (MIs). The BSA follows $B\,S\,A\,\left(m^{2}\right)=\sqrt{\frac{H\,t\,\left(\mathrm{cm}\right)*W\,t\,\left(\mathrm{kg}\right)}{3600}}$ [11].

### 2.3 Statistical Analysis

Missing values and outliers were addressed using multiple imputations via the Multivariate Imputation by Chained Equations (MICE) package [17]. As the database was systematically monitored by a data committee, missing values and outliers represented less than 2% of all metrics. We assumed that missing data and misrecordings occurred randomly [18], and used predictive mean matching to generate five imputed datasets suitable for logistic model fitting [19]. For multivariate logistic regression, we adjusted for the following variables based on clinical expertise: age, gender, smoking within two weeks prior to surgery, diabetes, hypertension, hyperlipidemia, the last serum creatinine test before surgery, the last total cholesterol test, the last low-density lipoprotein test, the last blood glucose test, preoperative eGFR, and history of cerebrovascular events. We used weight-of-Evidence binning which is a technique for binning both continuous and categorical independent variables in a way that provides the most robust bifurcation of the data against the dependent variable. This technique was implemented by the woebin function from R. Continuous variables were categorized according to established cutoffs. The bLVEF was optimally binned using evidence-based segmentation via the scorecard package (https://CRAN.R-project.org/package=scorecard), and coefficients were calculated using Spearman correction. Continuous variables are presented as mean ± standard deviation and were compared between groups using one-way Analysis of Variance (ANOVA). Categorical variables are presented as counts (percentages) and were compared between groups using Pearson’s chi-square test or Fisher’s exact test, as appropriate. The Cochran-Armitage trend test was used to assess trends across ordered LVEF categories. The area under the curve (AUC) for the receiver operating characteristics was assessed using the DeLong method. Using the generalized additive model to evaluate the nonlinear relationship. Sample size calculation indicated that 695 patients with bLVEF <85 would provide 99.50% power to detect a minimum clinically meaningful mortality rate of 5.18%, with a two-sided alpha of 0.05, compared to patients with bLVEF ≥85. All analyses were performed using R version 3.4.2 (The R Foundation for Statistical Computing, Vienna, Austria) (http://www.r-project.org/).

## 3. Results

### 3.1 Baseline

A total of 7927 patients were included in the final analysis, among whom 7093 (89.48%) had normal LVEF, 639 (8.06%) had HFmrEF, and 195 (2.46%) had HFrEF. The cohort’s mean age was 62.61 ± 8.70 years consisting of 6051 (76.33%) males and 3573 (45.07%) current or former smokers (Table 1). As expected, HFrEF or HFmrEF exhibited higher rates of comorbidities, including diabetes mellitus, abnormal serum creatinine, glucose and eGFR (*p* < 0.01 for trend), as well as higher previous myocardial infarction rates (Table 1, *p* for trend <0.01). Patients with low LVEF also had higher LVEDd and LAD, poorer New York Heart Association Functional Classification, and lower statins use (*p* for trend = 0.07). However, the use of Statins, ACEI/ARB did not differ significantly from patients with normal range LVEF (Table 1). These findings suggest that patients with reduced LVEF have a poorer preoperative baseline condition compared to those with normal LVEF. Baseline characteristics stratified by bLVEF are provided in the **Supplementary Material**.

**Table 1.** Patient characteristics according to LVEF category*.

		Total	LVEF <40	40 ≤ LVEF ≤ 49	LVEF ≥50	*p-*value
Number		7927	195	639	7093	
Age		62.61 ± 8.70	60.26 ± 9.43	61.72 ± 9.04	62.75 ± 8.63	<0.01
Gender (male)-n (%)		6051 (76.33%)	170 (87.18%)	542 (84.82%)	5339 (75.27%)	<0.01
BMI		25.69 ± 3.15	25.26 ± 3.02	25.5 ± 3.16	25.71 ± 3.16	0.05
BSA		1.82 ± 0.17	1.84 ± 0.16	1.83 ± 0.16	1.82 ± 0.17	0.10
Smoking-n (%)		3573 (45.07%)	116 (59.49%)	336 (52.58%)	3121 (44.00%)	<0.01
Diabetes-n (%)		3103 (39.14%)	94 (48.21%)	302 (47.26%)	2707 (38.16%)	<0.01
Hypertension-n (%)		4997 (63.04%)	102 (52.31%)	386 (60.41%)	4509 (63.57%)	<0.01
Hyperlipidemia-n (%)		2698 (34.04%)	68 (34.87%)	215 (33.65%)	2415 (34.05%)	0.96
Past medical history						
	Peripheral vascular disease-n (%)	240 (3.03%)	7 (3.59%)	19 (2.97%)	214 (3.02%)	0.89
	Previous cerebrovascular event-n (%)	1058 (13.35%)	25 (12.82%)	94 (14.71%)	939 (13.24%)	0.58
	Previous MI-n (%)	1256 (15.84%)	82 (42.05%)	205 (32.08%)	969 (13.66%)	<0.01
	Previous PCI-n (%)	1060 (13.37%)	26 (13.33%)	108 (16.9%)	926 (13.06%)	0.02
	NYHA1-n (%)	6087 (76.79%)	152 (78.35%)	497 (77.78%)	5438 (76.66%)	<0.01
	NYHA2-n (%)	4489 (56.63%)	96 (49.48%)	336 (52.58%)	4053 (57.13%)	
	NYHA3-n (%)	1518 (19.15%)	47 (24.23%)	144 (22.54%)	1327 (18.71%)	
	NYHA4-n (%)	82 (1.03%)	9 (4.64%)	15 (2.35%)	58 (0.82%)	
Last blood tests before surgery						
	Serum creatinine (µmol/L)	74.06 ± 20.90	84.54 ± 29.80	80.08 ± 25.68	73.23 ± 19.93	<0.01
	Serum total cholesterol (mmol/L)	4.00 ± 0.98	4.05 ± 1.05	3.94 ± 1.00	4.01 ± 0.98	0.71
	Serum low-density lipoprotein	2.37 ± 0.82	2.44 ± 0.90	2.37 ± 0.84	2.37 ± 0.81	0.53
	eGFR (mL/min/1.73 m^2^)	95.45 ± 11.22	92.72 ± 13.01	93.44 ± 12.29	95.70 ± 11.04	<0.01
	Blood glucose (mmol/L)	6.49 ± 2.07	6.76 ± 2.04	6.68 ± 1.97	6.46 ± 2.07	<0.01
Ultrasound indicators						
	LVEDd (mm)	48.98 ± 5.59	58.31 ± 6.88	55.27 ± 6.13	48.16 ± 4.86	<0.01
	LAD (mm)	35.86 ± 7.61	39.04 ± 7.04	38.29 ± 8.11	35.55 ± 7.52	<0.01
	LVEF (%)	60.24 ± 8.51	36.20 ± 2.16	44.32 ± 2.88	62.34 ± 6.08	<0.01
	Normalized by weight/100	43.22 ± 8.91	26.17 ± 4.31	32.03 ± 5.26	44.7 ± 8.01	<0.01
	Normalized by BMI/100	15.48 ± 2.91	9.17 ± 1.23	11.31 ± 1.57	16.03 ± 2.50	<0.01
	Normalized by BSA	109.63 ± 18.16	66.49 ± 7.39	81.24 ± 8.93	113.37 ± 14.90	<0.01
Preoperative medication						
	Nitrate lipid drugs-n (%)	1733 (21.86%)	41 (21.03%)	130 (20.34%)	1562 (22.02%)	0.60
	Catecholamines-n (%)	30 (0.38%)	1 (0.51%)	3 (0.47%)	26 (0.37%)	0.62
	β-blockers-n (%)	6611 (83.40%)	149 (76.41%)	546 (85.45%)	5916 (83.41%)	0.01
	ACEI or ARB-n (%)	1571 (19.82%)	37 (18.97%)	129 (20.19%)	1405 (19.81%)	0.94
	Statins-n (%)	5236 (66.05%)	115 (58.97%)	413 (64.63%)	4708 (66.38%)	0.07
	Aspirin-n (%)	2284 (28.81%)	58 (29.74%)	186 (29.11%)	2040 (28.76%)	0.94
	Clopidogrel-n (%)	555 (7.00%)	11 (5.64%)	38 (5.95%)	506 (7.13%)	0.40
	Ticagrelor-n (%)	399 (5.06%)	13 (6.67%)	33 (5.19%)	353 (4.98%)	0.56

BMI, body mass index; BSA, body surface area; NYHA, New York Heart Association; MI, myocardial infarction; PCI, percutaneous coronary intervention; eGFR, estimated glomerular filtration rate; LVEF, left ventricular ejection fraction; LVEDd, left ventricular end-diastolic diameter; LAD, left atrial dimension; ACEI, angiotensin-converting enzyme inhibitor; ARB, angiotensin receptor blocker. *Smoking within two weeks before surgery. Serum creatinine, serum total cholesterol, serum low-density lipoprotein, eGFR, blood glucose, LVEF, LVEDd, and LAD are the last tests before surgery. Nitrate lipid drugs are administered intravenously 24 hours before surgery. Catecholamines are administered intravenously 48 hours before surgery. β-blockers and statins are administered orally 24 hours before surgery. ACEI or ARB is administered orally 48 hours before surgery. Aspirin, clopidogrel, and ticagrelor are administered orally 5 days before surgery.

### 3.2 HFrEF and HFmrEF LVEF Is a Negative Prognostic Factor 

**Fig. 1.**
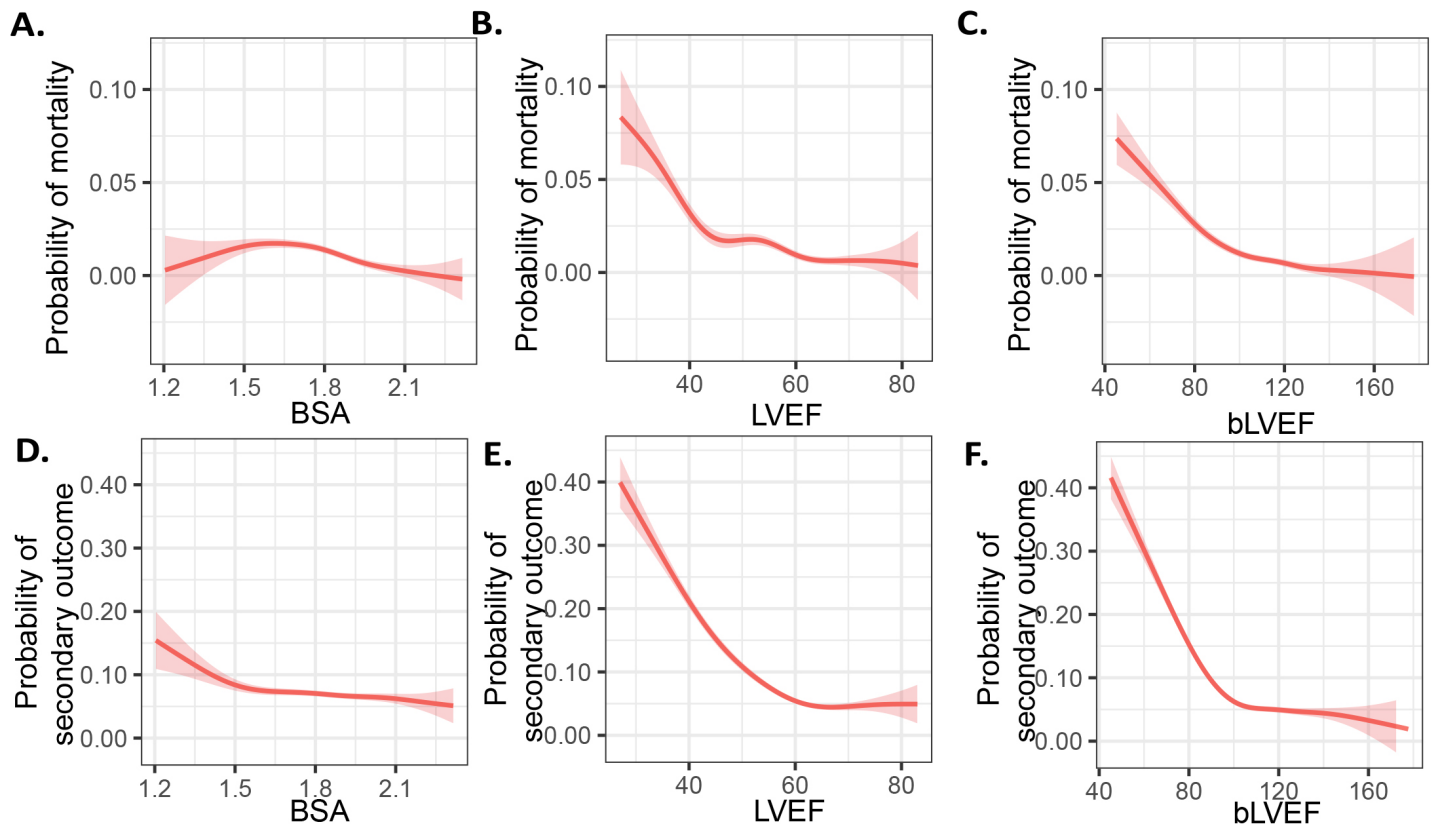
**BSA, LVEF and bLVEF for primary and secondary endpoints**. (A–C) Probability of mortality and (D–F) adverse events using a generalized additive model. LVEF, left ventricular ejection fraction; BSA, body surface area; bLVEF, BSA-weighted LVEF.

We further analyzed outcomes across LVEF categories. As shown in Table 2, HFrEF and HFmrEF patients experienced higher rates of multiorgan failure, acute kidney injury (AKI), and the cumulative mechanical ventilation time (Table 2, *p* for trend < 0.01). Mortality, intensive care unit (ICU) length of stay, use of IABP, and use of ECMO increased with decreasing LVEF (*p* for trend = 0.05), while postoperative stroke (*p* for trend = 0.88), postoperative MI (*p* for trend = 0.52) and re-admission to ICU (*p* for trend = 0.51) did not show statistical differences (Table 2). Multivariable regression analysis identified HFrEF (adjusted odds ratio [OR] 6.50, 95% confidence interval [CI] 3.02–12.68, *p* < 0.01, Table 3, Fig. 1B) and HFmrEF as significant negative predictors of survival, suggesting that an increase in the odds of mortality compared to the reference group (LVEF ≥50%). Likewise, patients with lower LVEF experienced a greater incidence of adverse events (adjusted OR 6.98, 95% CI 4.96–9.72, *p* < 0.01, Table 3). These findings underscore low LVEF as a significant negative prognostic factor for both postoperative survival and adverse outcomes.

**Table 2.** Patient outcomes according to LVEF category*.

	Total	LVEF <40	40 ≤ LVEF ≤ 49	LVEF ≥50	*p-*value
Number	7927	195	639	7093	
Perioperative blood transfusion-n (%)	5183 (65.38%)	135 (69.23%)	429 (67.14%)	4619 (65.12%)	0.31
Mechanical ventilation duration (hour)	23.55 ± 23.34	37.39 ± 32.48	28.48 ± 28.63	22.73 ± 22.32	<0.01
Initial ICU length of stay (hour)	31.50 ± 31.83	51.81 ± 46.01	39.97 ± 38.32	30.18 ± 30.38	<0.01
Perioperative blood loss (mL)	1017.85 ± 863.88	1011.68 ± 897.15	1079.30 ± 906.90	1012.48 ± 858.88	<0.01
Serum creatinine (µmol/L)	84.57 ± 31.47	97.80 ± 42.61	91.89 ± 37.23	83.55 ± 30.36	<0.01
eGFR (mL/min/1.73 m^2^)	94.28 ± 29.85	85.03 ± 33.05	88.18 ± 30.64	95.09 ± 29.58	<0.01
AKI-n (%)	641 (8.09%)	22 (11.28%)	64 (10.02%)	555 (7.82%)	0.04
Use of IAPB-n (%)	450 (5.68%)	55 (28.21%)	80 (12.52%)	315 (4.44%)	<0.01
Use of ECMO-n (%)	37 (0.47%)	2 (1.03%)	6 (0.94%)	29 (0.41%)	0.05
Reoperation-n (%)	122 (1.54%)	5 (2.56%)	10 (1.56%)	107 (1.51%)	0.41
Postoperative MI-n (%)	48 (0.61%)	2 (1.03%)	4 (0.63%)	42 (0.59%)	0.52
Postoperative stroke-n (%)	64 (0.81%)	1 (0.51%)	6 (0.94%)	57 (0.80%)	0.88
Re-intubation-n (%)	65 (0.82%)	3 (1.54%)	9 (1.41%)	53 (0.75%)	0.08
Re-enter ICU-n (%)	132 (1.67%)	5 (2.56%)	11 (1.72%)	116 (1.64%)	0.51
Multiorgan failure-n (%)	45 (0.57%)	6 (3.08%)	11 (1.72%)	28 (0.39%)	<0.01
Dead-n (%)	68 (1.05%)	9 (5.49%)	10 (1.96%)	49 (0.84%)	<0.01

LVEF, left ventricular ejection fraction; ICU, intensive care unit; eGFR,estimated glomerular filtration rate; AKI, acute kidney injury; IABP, intra-aortic balloon pump; ECMO, extracorporeal membrane oxygenation; MI, myocardial infarction. *Serum creatinine is the maximum serum creatinine after surgery; eGFR is the minimum eGFR after surgery.

**Table 3.** **Unadjusted and adjusted logistic regression model of the association between bLVEF, LVEF, and BSA with prognosis of patients**.

		Mortality					Secondary outcomes				
		Univariate		Multivariate		AUC	Univariate		Multivariate		AUC
		OR	*p*-value	OR	*p-*value		OR	*p*-value	OR	*p-*value	
Numerical bLVEF		0.96 (0.95~0.97)	<0.01	0.97 (0.96~0.98)	<0.01	0.69	0.97 (0.97~0.98)	<0.01	0.97 (0.97~0.98)	<0.01	0.63
Categorized bLVEF		0.47 (0.38~0.58)	<0.01	0.50 (0.40~0.62)	<0.01	0.70	0.59 (0.54~0.64)	<0.01	0.59 (0.54~0.65)	<0.01	0.64
	<85										
	[85, 120)	0.40 (0.23~0.71)	<0.01	0.41 (0.23~0.73)	<0.01		0.38 (0.29~0.50)	<0.01	0.39 (0.30~0.51)	<0.01	
	[120, 135)	0.25 (0.15~0.42)	<0.01	0.27 (0.16~0.46)	<0.01		0.26 (0.20~0.32)	<0.01	0.26 (0.21~0.33)	<0.01	
	[135, INF)	0.08 (0.03~0.17)	<0.01	0.10 (0.04~0.22)	<0.01		0.21 (0.16~0.27)	<0.01	0.21 (0.16~0.28)	<0.01	
Numerical LVEF		0.94 (0.92~0.96)	<0.01	0.94 (0.92~0.96)	<0.01	0.66	0.94 (0.93~0.95)	<0.01	0.94 (0.93~0.95)	<0.01	0.64
Categorized LVEF		2.37 (1.71~3.20)	<0.01	2.48 (1.77~3.40)	<0.01	0.64	2.65 (2.30~3.05)	<0.01	2.65 (2.29~3.06)	<0.01	0.63
	[50, INF)										
	[40, 50)	2.21 (1.25~3.70)	<0.01	2.30 (1.29~3.91)	<0.01		2.71 (2.16~3.36)	<0.01	2.68 (2.13~3.34)	<0.01	
	<40	5.88 (2.80~11.14)	<0.01	6.50 (3.02~12.68)	<0.01		6.89 (4.93~9.50)	<0.01	6.98 (4.96~9.72)	<0.01	
Numerical BSA		0.10 (0.03~0.33)	<0.01	0.21 (0.05~0.99)	0.05	0.63	0.58 (0.35~0.97)	0.04	0.54 (0.29~1.01)	0.05	0.52
Categorized BSA		0.70 (0.60~0.82)	<0.01	0.76 (0.62~0.91)	<0.01	0.63	0.94 (0.88~10.00)	0.04	0.93 (0.86~10.00)	0.05	0.53
	<1.68										
	[1.68, 1.79)	1.12 (0.65~1.91)	0.69	1.30 (0.73~2.32)	0.37		0.93 (0.72~1.21)	0.58	0.88 (0.67~1.16)	0.37	
	[1.79, 1.87)	0.73 (0.40~1.31)	0.29	0.90 (0.46~1.73)	0.75		0.85 (0.65~1.11)	0.22	0.79 (0.58~1.06)	0.11	
	[1.87, 1.97)	0.38 (0.17~0.77)	<0.01	0.52 (0.22~1.14)	0.11		0.81 (0.61~1.06)	0.12	0.76 (0.56~1.03)	0.08	
	[1.97, INF)	0.22 (0.08~0.51)	<0.01	0.33 (0.11~0.82)	0.02		0.78 (0.59~1.02)	0.07	0.74 (0.54~1.02)	0.07	

LVEF, left ventricular ejection fraction; BSA, body surface area; bLVEF, BSA-weighted LVEF; AUC, area under the curve; eGFR, estimated glomerular filtration rate; INF, infinity. Age, gender, smoking within two weeks before surgery, diabetes, hypertension, hyperlipidemia, last test of serum creatinine before surgery, last test of serum total cholesterol before surgery, last test of serum low-density lipoprotein before surgery, last test of blood glucose before surgery, use of cardiopulmonary bypass (CPB), preoperative eGFR, and previous cerebrovascular events were used for the multivariate regression. bLVEF was categorized into 4 groups based on a weight of tree-like segmentation binning.

### 3.3 LVEF Normalized for BSA

The mean BSA is 1.82 ± 0.17 m^2^, which showed a slightly negative relationship with the LVEF (R = –0.05, *p* < 0.01, **Supplementary Fig. 2**) and this also reached statistical difference when compared to 3 LVEF groups (*p* for trend = 0.03). As anticipated, a high BSA was linked to decreased mortality (adjusted OR 0.76, 95% CI 0.62–0.91, *p* < 0.01, Table 3, Fig. 1A) as well as secondary outcomes (adjusted OR 0.93, 95% CI 0.86–1, *p* = 0.05, Table 3, Fig. 1D). Given that LVEF has not been previously analyzed in conjunction with BSA, we hypothesized that bLVEF, which is defined as LVEF multiplied by BSA, may serve as a more effective predictor of postoperative prognosis. We found that the bLVEF decreased with LVEF (*p* for trend < 0.01, Fig. 1, **Supplementary Tables 2,3**), but revealed a different relationship with the mortality than LVEF alone, that is, a plateau appeared near to the 85% of bLVEF.

### 3.4 The bLVEF Is a Better Predictor of Mortality

To evaluate bLVEF as a predictor of postoperative mortality and adverse events, as well as to compare its predictive efficacy with other outcome predictors, we performed univariate and multivariate logistic regression analyses. Our results indicated a significant correlation between bLVEF and postoperative mortality, with findings showing an adjusted OR of 0.97 (95% CI: 0.96–0.98, *p* < 0.01) for mortality risk (refer to Table 3 and Fig. 1C). Additionally, bLVEF was strongly linked to secondary outcomes, revealing an adjusted OR of 0.97 (95% CI: 0.97–0.98, *p* < 0.01 see Fig. 1D,E), which exhibited a consistent trend alongside decreases in bLVEF levels (see Table 3 and Fig. 1F). These results imply that bLVEF is a significant negative predictor for both mortality and adverse postoperative events.

**Fig. 2.**
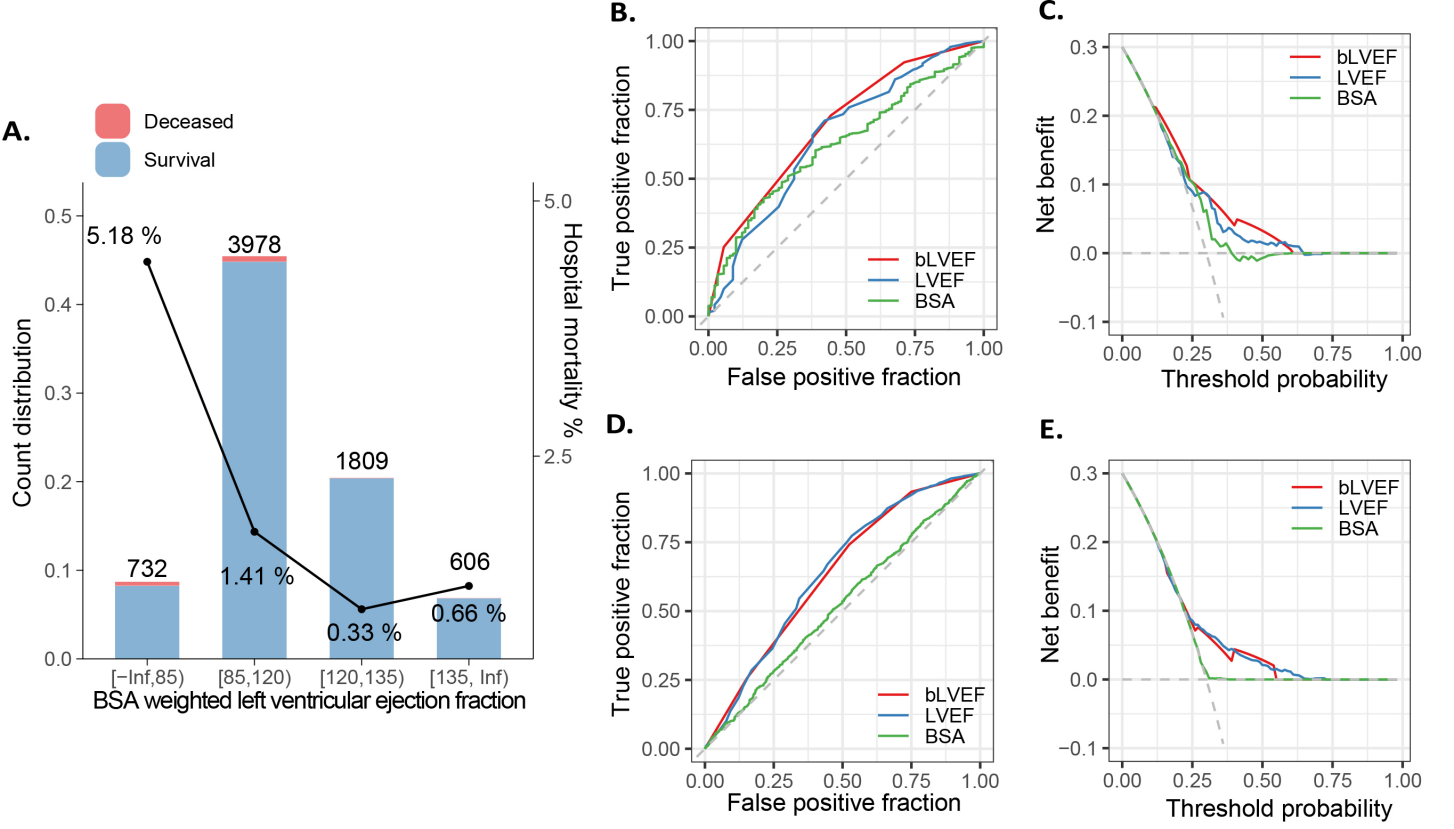
**Segmentation of bLVEF and its abilities to predict clinical outcome**. (A) Supervised tree-like segmentation of bLVEF (B) ROC and (C) decision curve analysis for mortality, and (D,E) secondary outcomes. bLVEF, BSA-weighted LVEF; LVEF, left ventricular ejection fraction; BSA, body surface area; ROC, receiver operating characteristic.

To enhance the practicality of bLVEF in clinical applications, we employed a tree-based segmentation method to categorize bLVEF into discrete ranges. Our analysis revealed four distinct categories: [0, 85), [85, 120), [120, 135), and [135, ∞) (adjusted K-S statistic of 0.42, *p* = 0.01, see Fig. 2A and **Supplementary Fig. 3**). The categorical version of bLVEF demonstrated comparable discrimination abilities to its numerical counterpart in predicting mortality, achieving an AUC of 0.70 (*p* < 0.01). Notably, among the variables analyzed, bLVEF emerged as the most significant predictor of mortality, surpassing both BSA and LVEF. While BSA (AUC 0.63) and LVEF (AUC 0.64) showed some association with mortality risk, their AUC values were lower and less significant compared to bLVEF (DeLong test, *p* < 0.01; see Fig. 2B,C). We propose to use the 85 as the threshold to categorize the patients and high-risk and low-risk groups. We added the relevant description to the manuscript. Importantly, patients with a bLVEF of less than 85 exhibited a substantially higher risk of mortality compared to those with a bLVEF of 85 or greater, with an OR of 4.65 (95% CI: 3.81–5.83, *p* < 0.01; see Fig. 2B,C). This suggests that a bLVEF threshold of less than 85 is a more reliable indicator of high-risk patients than LVEF or BSA alone. For the secondary outcome, there was no statistically significant difference in the AUC between LVDD and BLVDD (DeLong test, *p* = 0.5661, see Fig. 2D,E).

## 4. Discussion

In this multi-center cohort investigation, we found that (1) low LVEF is a significant predictor of postoperative survival and adverse events; (2) BSA exhibited a slight negative correlation with LVEF; (3) bLVEF emerged as the most consistent predictor of mortality compared to BSA and LVEF; and (4) a bLVEF cutoff of 85 effectively identified patients at high risk of mortality.

Cardiac surgery improves survival in patients with advanced left ventricular dysfunction compared to medical management [20]. However, low preoperative LVEF is a known risk factor for the adverse outcomes following CABG [21]. Since Benetti *et al*. [2] successfully performed OPCABG with the emphasis on myocardial protection and the surgeon’s increasing proficiency in bypass grafting, OPCABG has become a well-developed and safe procedure [22]. Recent years have seen increased interest in its use for severe coronary artery disease, with several studies highlighting its efficacy in patients with low LVEF [23]. Shennib *et al*. [24] reported favorable outcomes and low mortality rates in patients with impaired ventricular function undergoing off-pump surgical revascularization. Arom *et al*. [22] discovered that performing multivessel coronary artery bypass using the OPCAB technique is both suitable and feasible for patients with left ventricular function at or below 30%. In addition, Ueki *et al*. [10] reported that OPCABG is linked to significantly lower rates of early mortality and morbidity in patients with an ejection fraction of less than 30%. Despite these findings, comparative studies on OPCABG across different EF categories remain limited. In our cohort, 7093 (89.48%) patients had normal LVEF, 639 (8.06%) had HFmrEF, and 195 (2.46%) had HFrEF, consistent with previously reported population distributions [25]. Patients with HFrEF or HFmrEF exhibited higher rates of diabetes, hypertension, impaired kidney function, and prior myocardial infarctions, along with elevated LVEDd and LAD, worse NYHA Functional Classification scores, and greater statin use (*p* for trend < 0.01). These findings highlight the poorer preoperative baseline condition of patients with low LVEF compared to those with normal LVEF.

CAD with reduced EF presents a significant challenge to OPCABG. Proper exposure of the anastomosis site during OPCABG necessitates cardiac manipulation, which can compress the left ventricular outflow tract and lead to hypotension. In patients with low LVEF, the limited cardiac reserve makes them highly susceptible to abrupt drops in blood pressure. Additionally, patients with left main trunk and three-vessel disease are at increased risk of malignant arrhythmias during cardiac manipulation [4, 26, 27]. Therefore, it is necessary to implement appropriate emergency measures and coordinate with anesthesiologists before repositioning the heart. The use of IABP helps to improve the success rate of OPCABG surgery [28]. Suzuki *et al*. [29] indicated that preoperative IABP therapy in high-risk coronary patients effectively prevents hemodynamic instability and yields surgical outcomes similar to those seen in moderate to low-risk patients. In our study, HFrEF and HFmrEF were identified as strong negative prognostic factors for mortality, suggesting that a decrease in the odds of mortality compared to the reference group (LVEF ≥50%). Similarly, adverse event rates rose by 63% with each LVEF category reduction. Surgeons must carefully assess intraoperative conditions to determine whether to proceed with OPCABG. If cardiac function deteriorates significantly during the procedure, transitioning to on-pump coronary artery bypass grafting should be considered to mitigate further risks.

Echocardiography is extensively utilized for diagnosing and managing cardiac conditions, particularly in the preoperative assessment of patients undergoing cardiac surgery [30, 31]. However, cardiac surgeons often rely on unstandardized absolute values when interpreting echocardiographic parameters, despite the influence of body mass factors on heart structure [32]. Assessing echocardiographic indicators solely based on absolute values does not enable precise diagnosis of cardiac conditions [33]. BSA, a metric for characterizing body size, is frequently employed to normalize mass and volume and to index physiological parameters related to cardiovascular disease [34, 35]. A study has indicated that left ventricle (LV) diameter provides a basic and simplified evaluation of a three-dimensional structure, failing to account for the more intricate variations in ventricular shape or size [36]. To address this, the American Society of Echocardiography and the European Association of Cardiovascular Imaging recommended indexing echocardiographic parameters, such as right and left ventricular sizes, to BSA [35]. Moreover, the Simpson’s biplane method, which uses orthogonal long-axis views, allows for a more accurate calculation of LV volume that can be indexed to BSA for improved precision [37]. LVEF is a parameter obtained from echocardiography that measures the amount of blood ejected from the heart’s left ventricle—the primary pumping chamber—with each contraction [38]. Furthermore, LVEF is acknowledged as an independent risk factor for CABG. A lower ejection fraction correlates with increased perioperative mortality and reduced five-year survival rates [39, 40]. While the conventional definition of the normal range for EF is derived from the general population, encompassing groups with varying clinical characteristics and prognoses. Thuijs DJFM *et al*. [25] found a U-shaped association between LVEF and all-cause mortality three years after CABG. In our study, we identified a J-shaped curve in LVEF when predicting perioperative mortality in patients undergoing OPCABG. This observation is of considerable clinical significance, as it suggests a potential for misinterpretation among cardiac clinicians. Specifically, clinicians may incorrectly assume that patients with an LVEF at the relatively “good” level have adequate cardiac function, thus deeming them to be at a low risk for perioperative complications. However, this oversimplification can obscure the true risks associated with the procedure. Given the complexity of heart failure and the multitude of factors influencing perioperative outcomes, it is imperative to consider all relevant risk factors beyond just LVEF when making surgical decisions for patients undergoing OPCABG. Relying solely on LVEF could lead to incomplete assessments of a patient’s functional status, potentially compromising patient safety. To mitigate this risk of bias, we advocate for the normalization of LVEF to BSA, a practice that enhances the reliability of prognostic assessments. By doing so, we established a critical numerical threshold of 85 as a ‘divider’ that serves to refine the prediction of perioperative mortality. This threshold represents a key parameter in stratifying patient risk, enabling more accurate and personalized surgical decision-making. It is worth noting that the observed patterns are hypothesis-generating and require validation in cohorts with harmonized analytical frameworks. Ultimately, our findings underscore the importance of incorporating LVEF normalization into clinical practice, ensuring that all relevant factors are weighed appropriately to optimize patient outcomes in OPCABG. This approach can significantly improve the predictive accuracy regarding mortality and enhance the overall management of patients undergoing coronary revascularization.

There are limitations in our study. Firstly, the retrospective cohort design relies on historical patient data, which restricts the availability of comprehensive preoperative activity tolerance information. Additionally, the extended follow-up period resulted in a considerable loss to follow-up. Secondly, common post-surgery complications such as severe heart failure and atrial fibrillation were not included due to challenges in accurately documenting them during follow-up assessments. Thirdly, the study did not discuss gender differences, which will be further explored in future research. Lastly, body surface area is calculated using a formula based on weight and height, which does not reflect the true surface area of an individual. Furthermore, factors like age, gender, and race may confound the BSA measurement, highlighting the need for future research to investigate the correlating variables associated with BSA.

## 5. Conclusions

bLVEF serves as a key predictor of mortality in OPCABG, effectively eliminating BSA-related bias and demonstrating a strong capacity to predict mortality.

## Data Availability

All data reported in this paper will be shared by the lead contact upon request.

## Abbreviations

CABG, coronary artery bypass grafting; OPCABG, off-pump coronary artery bypass grafting; HF, heart failure; LVEF, left ventricular ejection fraction; BMI, body mass index; BSA, body surface area; GFR, glomerular filtration rate; HFrEF, heart failure with reduced ejection fraction; HFmrEF, heart failure with mid-range ejection fraction; LVEDd, left ventricular end-diastolic diameter; LAD, left atrial dimension; ACEI, angiotensin-converting enzyme inhibitor; ARB, angiotensin receptor blocker; ECMO, extracorporeal membrane oxygenation; IABP, intra-aortic balloon pump; ICU, intensive care unit; MSA, multiple system atrophy; AKI, acute kidney injury; MI, myocardial infarction; AUC, area under the curve.

## Supplementary Material

Supplementary material associated with this article can be found, in the online version, at https://doi.org/10.31083/RCM26681.
