# Supplementary material for: Body Surface Area-Weighted Left Ventricular Ejection Fraction Enhances Prediction Accuracy of OPCABG Outcomes: A Large Multi-Center Cohort Study
Source: Rev Cardiovasc Med. 2025 Nov 26;26(11):26681. doi: 10.31083/RCM26681 (PMC12680993; doi:10.31083/RCM26681)
Supplement: Supplementary file 1 [file 2153-8174-26-11-26681-s1.docx]

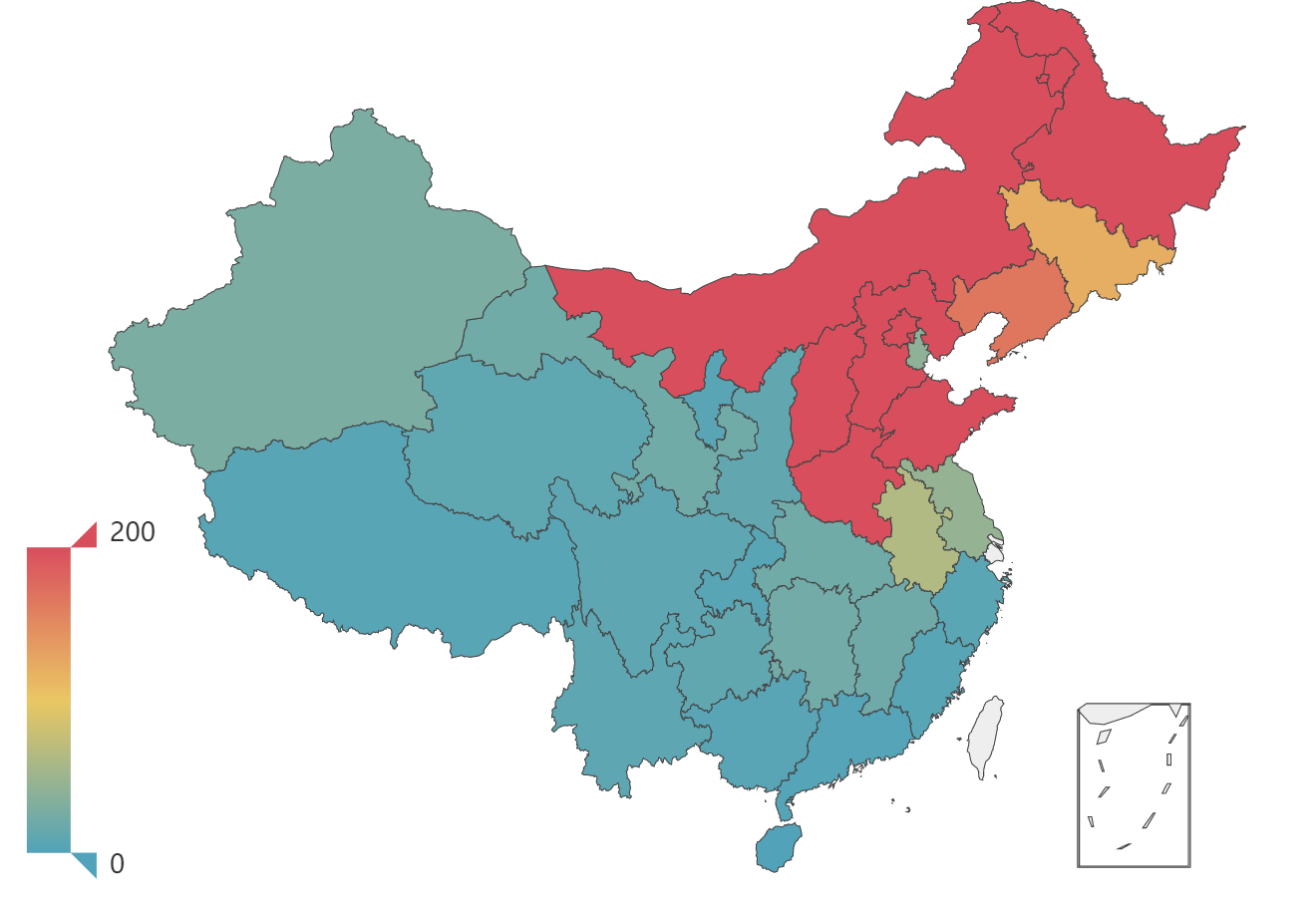


**Supplementary Fig. 1. Regional distribution of enrolled patients.**


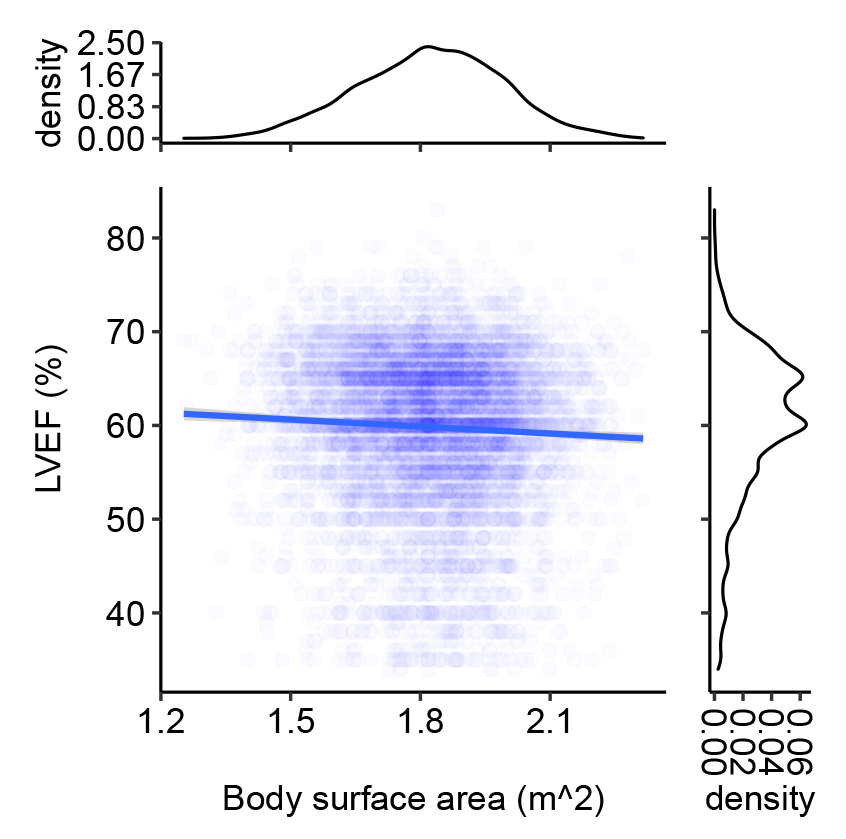


**Supplementary Fig. 2. Scatter plot and density distribution of LVEF and BSA.**


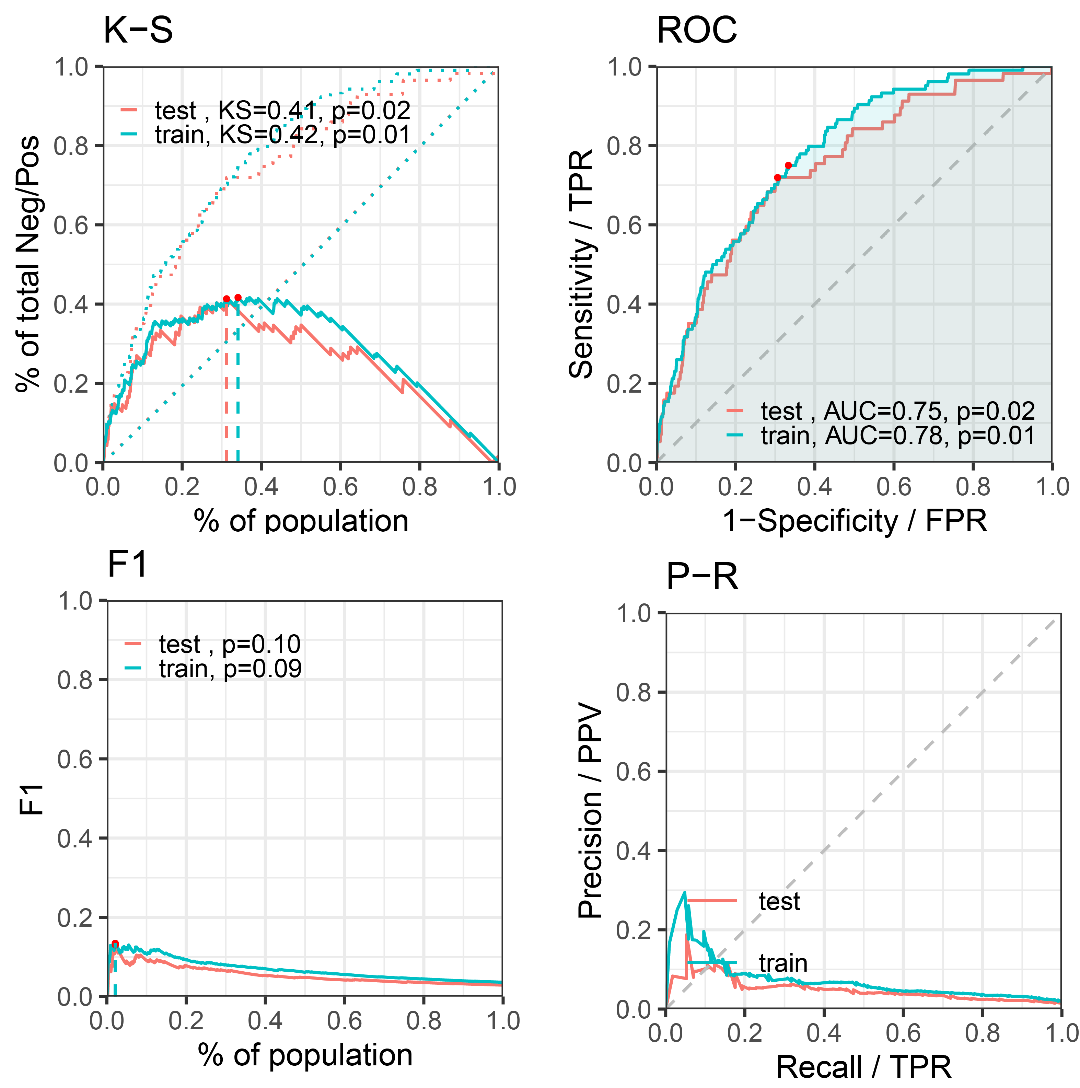


**Supplementary Fig. 3. Supervised tree-like segmentation of bLVEF and its evaluation and validation.**

(A) K-S, (B) ROC, (C) F1and (D) PR curves when comparing train and test sets.

Supplementary Table 1 Patient characteristics according to bLVEF category*

|  | **Total** | **bLVEF＜110** | **bLVEF≥110** | ***P-*value** |
| --- | --- | --- | --- | --- |
| **Number** | 7927 | 3798 | 4129 |  |
| **Age** | 62.61±8.70 | 62.36±8.97 | 62.92±8.65 | <0.01 |
| **Gender (male)** | 6051(76.33%) | 2548(67.09%) | 3503(84.77%) | <0.01 |
| **BMI** | 25.69±3.15 | 24.60±2.98 | 26.68±2.97 | <0.01 |
| **BSA** | 1.82±0.17 | 1.74±0.16 | 1.89±0.14 | <0.01 |
| **Smoking** | 3573(45.07%) | 1546(40.68%) | 2027(49.09%) | <0.01 |
| **Diabetes** | 3103(39.14%) | 1589(41.79%) | 1514(36.64%) | <0.01 |
| **Hypertension** | 4997(63.04%) | 2289(60.24%) | 2708(65.58%) | <0.01 |
| **Hyperlipidemia** | 2698(34.04%) | 1276(33.60%) | 1422(34.49%) | 0.40 |
| **Past medical history** |  |  |  |  |
| **Peripheral vascular disease** | 240(3.03%) | 115(3.00%) | 125(3.00%) | 0.99 |
| **Previous cerebrovascular event** | 1058(13.35%) | 515(13.56%) | 543(13.15%) | 0.59 |
| **Previous MI** | 1256(15.84%) | 776(20.43%) | 480(11.63%) | <0.01 |
| **Previous PCI** | 1060(13.37%) | 511(13.45%) | 549(13.3%) | 0.84 |
| **NYHA1** | 6089(76.81%) | 2895(76.22%) | 3194(77.28%) | <0.01 |
| **NYHA2** | 4489(56.63%) | 2090(55.03%) | 2397(58.05%) |  |
| **NYHA3** | 1518(19.15%) | 751(19.77%) | 766(18.55%) |  |
| **NYHA4** | 82(1.03%) | 54(1.42%) | 28(0.68%) |  |
| **Last blood tests before surgery** |  |  |  |  |
| **Serum creatinine (umol/L)** | 74.06±20.90 | 73.35±21.47 | 74.19±17.91 | 0.06 |
| **Serum total cholesterol (mmol/L)** | 4±0.98 | 4.06±0.99 | 3.96±0.98 | <0.01 |
| **Serum low-density lipoprotein** | 2.37±0.82 | 2.40±0.84 | 2.36±0.80 | 0.01 |
| **eGFR (mL/min/1.73m^2^)** | 95.45±11.22 | 95.49±11.71 | 95.53±10.56 | 0.88 |
| **Blood glucose (mmol/L)** | 6.49±2.07 | 6.54±1.99 | 6.45±2.16 | 0.06 |
| **Ultrasound indicators** |  |  |  |  |
| **LVDd (mm)** | 48.98±5.59 | 49.84±6.45 | 48.23±4.50 | <0.01 |
| **LAD (mm)** | 35.86±7.61 | 35.97±7.52 | 35.62±7.86 | 0.46 |
| **LVEF(%)** | 60.24±8.51 | 54.82±8.21 | 65.29±4.91 | <0.01 |
| Normalized by weight/100 | 43.22±8.91 | 36.12±5.51 | 49.77±5.93 | <0.01 |
| Normalized by BMI/100 | 15.48±2.91 | 13.42±2.22 | 17.38±2.06 | <0.01 |
| Normalized by BSA | 109.63±18.16 | 94.76±12.33 | 123.38±9.99 | <0.01 |
| **Preoperative medication** |  |  |  |  |
| **Nitrate lipid drugs** | 1736(21.9%) | 769(20.25%) | 967(23.37%) | <0.01 |
| **Catecholamines** | 30(0.38%) | 16(0.42%) | 14(0.36%) | 0.67 |
| **β-blockers** | 6611(83.4%) | 3159(83.18%) | 3452(83.65%) | 0.57 |
| **ACEI or ARB** | 1571(19.82%) | 834(21.95%) | 737(17.85%) | 0.20 |
| **Statins** | 5236(66.05%) | 2572(67.72%) | 2664(64.52%) | 0.12 |
| **Aspirin** | 2284(28.81%) | 1061(27.93%) | 1223(29.61%) | <0.01 |
| **Clopidogrel** | 555(7.00%) | 237(6.24%) | 318(7.70%) | 0.47 |
| **Ticagrelor** | 399(5.06%) | 170(4.47%) | 229(5.55%) | 0.96 |

Supplementary Table 2 Patient Characteristics According to bLVEF Category*

|  | **Total** | **bLVEF＜85** | **bLVEF≥85** | ***P-*value** |
| --- | --- | --- | --- | --- |
| **Number** | 7927 | 731 | 7196 |  |
| **Age** | 62.61±8.70 | 62.84±9.31 | 62.59±8.63 | <0.01 |
| **Gender (male)-n(%)** | 6051(76.33%) | 525(71.82%) | 5525(76.77%) | <0.01 |
| **BMI** | 25.69±3.15 | 24.17±3.15 | 25.84±3.11 | <0.01 |
| **BSA** | 1.82±0.17 | 1.73±0.18 | 1.89±0.17 | <0.01 |
| **Smoking-n(%)** | 3573(45.07%) | 330(45.14%) | 3243(45.07%) | <0.01 |
| **Diabetes-n(%)** | 3103(39.14%) | 345(47.19%) | 2757(38.31%) | <0.01 |
| **Hypertension-n(%)** | 4997(63.04%) | 409(55.87%) | 4589(63.76%) | <0.01 |
| **Hyperlipidemia-n(%)** | 2698(34.04%) | 229(31.28%) | 2472(34.35%) | 0.10 |
| **Past medical history** |  |  |  |  |
| **Peripheral vascular disease-n(%)** | 240(3.03%) | 25(3.42%) | 215(2.99%) | 0.68 |
| **Previous cerebrovascular event-n(%)** | 1058(13.35%) | 102(13.93%) | 958(13.31%) | 0.43 |
| **Previous MI-n(%)** | 1256(15.84%) | 238(32.51%) | 1018(14.14%) | <0.01 |
| **Previous PCI-n(%)** | 1060(13.37%) | 101(13.8%) | 959(13.32%) | 0.17 |
| **NYHA1-n(%)** | 6089(76.81%) | 581(79.37%) | 5507(76.52%) | <0.01 |
| **NYHA2-n(%)** | 4489(56.63%) | 384(52.46%) | 4104(57.02%) |  |
| **NYHA3-n(%)** | 1518(19.15%) | 172(23.5%) | 1346(18.7%) |  |
| **NYHA4-n(%)** | 82(1.03%) | 25(3.42%) | 57(0.79%) |  |
| **Last blood tests before surgery** |  |  |  |  |
| **Serum creatinine (umol/L)** | 74.06±20.90 | 78.13±24.92 | 73.35±19.03 | 0.56 |
| **Serum total cholesterol (mmol/L)** | 4±0.98 | 4.05±1.04 | 4.00±0.98 | <0.01 |
| **Serum low-density lipoprotein** | 2.37±0.82 | 2.42±0.88 | 2.37±0.81 | 0.01 |
| **eGFR (mL/min/1.73m^2^)** | 95.45±11.22 | 93.83±12.53 | 95.68±10.95 | 0.33 |
| **Blood glucose (mmol/L)** | 6.49±2.07 | 6.62±1.97 | 6.48±2.09 | <0.01 |
| **Ultrasound indicators** |  |  |  |  |
| **LVDd (mm)** | 48.98±5.59 | 54.6±7.16 | 48.44±5.06 | <0.01 |
| **LAD (mm)** | 35.86±7.61 | 37.41±8.17 | 35.62±7.65 | <0.01 |
| **LVEF(%)** | 60.24±8.51 | 43.14±5.99 | 62.01±6.57 | <0.01 |
| Normalized by weight/100 | 43.22±8.91 | 28.04±3.74 | 44.77±7.75 | <0.01 |
| Normalized by BMI/100 | 15.48±2.91 | 10.34±1.50 | 16.01±2.49 | <0.01 |
| Normalized by BSA | 109.63±18.16 | 74.11±7.56 | 113.28±14.68 | <0.01 |
| **Preoperative medication** |  |  |  |  |
| **Nitrate lipid drugs-n(%)** | 1736(21.9%) | 152(20.77%) | 1582(21.98%) | <0.01 |
| **Catecholamines-n(%)** | 30(0.38%) | 5(0.68%) | 26(0.36%) | 0.41 |
| **β-blockers-n(%)** | 6611(83.4%) | 603(82.38%) | 6012(83.53%) | 0.18 |
| **ACEI or ARB-n(%)** | 1571(19.82%) | 151(20.63%) | 1420(19.73%) | 0.05 |
| **Statins-n(%)** | 5236(66.05%) | 468(63.93%) | 4772(66.31%) | <0.01 |
| **Aspirin-n(%)** | 2284(28.81%) | 219(29.92%) | 2066(28.71%) | <0.01 |
| **Clopidogrel-n(%)** | 555(7.00%) | 46(6.28%) | 511(7.1%) | 0.06 |
| **Ticagrelor-n(%)** | 399(5.06%) | 46(6.33%) | 408(5.7%) | 0.53 |

BMI, body mass index; NYHA, New York Heart Association; MI, myocardial infarction; PCI, percutaneous coronary intervention; eGFR, estimated glomerular filtration rate; CABG, coronary artery bypass graft; LVEF, left ventricular ejection fraction; LVDd, left ventricular end-diastolic diameter; LAD, left atrial dimension; CPB, cardiopulmonary bypass; ACEI, ACE inhibitor; ARB, angiotensin receptor blocker.

*Smoking within two weeks before surgery.

*Serum creatinine, serum total cholesterol, Serum low-density lipoprotein, eGFR, Blood glucose, LVEF, LVDd, and LAD are the last tests before surgery.

*Nitrate lipid drugs are administered intravenously 24 hours before surgery.

*Catecholamines are administered intravenously 48 hours before surgery.

*β-blockers and statins are administered orally 24 hours before surgery.

*ACEI or ARB are administered orally 48 hours before surgery.

Supplementary Table 3 Patient Outcomes According to bLVEF Category*

|  | **Total** | **bLVEF＜85** | **bLVEF≥85** | ***P-*value** |
| --- | --- | --- | --- | --- |
| **Number** | 7927 | 731 | 7196 |  |
| **Perioperative blood transfusion-n(%)** | 5183(65.38%) | 511(69.81%) | 4674(64.94%) | <0.01 |
| **Mechanical ventilation duration (hour)** | 23.55±23.34 | 31.12±31.02 | 22.53±21.29 | <0.01 |
| **Initial ICU length of stay (hour)** | 31.50±31.83 | 42.72±41 | 30.16±30.03 | <0.01 |
| **Perioperative blood loss (ml)** | 1017.85±863.88 | 1441.27±746.34 | 1400.72±742.01 | <0.01 |
|  |  |  |  |  |
| **Serum creatinine (umol/L)** | 84.57±31.47 | 89.72±38.27 | 83.96±30.35 | 0.38 |
| **eGFR (mL/min/1.73m2)** | 94.28±29.85 | 91.12±33.04 | 94.62±29.46 | 0.05 |
| **AKI-n(%)** | 641(8.09%) | 73(9.97%) | 590(8.2%) | 0.10 |
|  |  |  |  |  |
| **Use of IAPB-n(%)** | 450(5.68%) | 125(17.08%) | 326(4.53%) | <0.01 |
| **Use of ECMO-n(%)** | 37(0.47%) | 6(0.82%) | 31(0.43%) | 0.02 |
| **Reoperation-n(%)** | 122(1.54%) | 12(1.64%) | 111(1.54%) | 0.54 |
| **Postoperative MI-n(%)** | 48(0.61%) | 7(0.96%) | 41(0.57%) | 0.17 |
| **Postoperative stroke-n(%)** | 64(0.81%) | 6(0.82%) | 58(0.81%) | 0.13 |
| **Re-intubation-n(%)** | 65(0.82%) | 12(1.64%) | 53(0.74%) | 0.02 |
| **Re-enter ICU-n(%)** | 132(1.67%) | 16(2.19%) | 117(1.63%) | 0.03 |
| **Multiorgan failure-n(%)** | 45(0.57%) | 16(2.19%) | 29(0.4%) | <0.01 |
| **Dead-n(%)** | 68(1.05%) | 21(3.69%) | 47(0.88%) | <0.01 |

*ICU, intensive care unit; eGFR, estimated glomerular filtration rate; BMI, body mass index; AKI, acute kidney injury; IABP, intra-aortic balloon pump; ECMO, extracorporeal membrane oxygenation; MI, myocardial infarction.

*Serum creatinine is the maximum serum creatinine after surgery; eGFR is the minimum eGFR after surgery.
